# 3D skeletal uptake of ^18^F sodium fluoride in PET/CT images is associated with overall survival in patients with prostate cancer

**DOI:** 10.1186/s13550-017-0264-5

**Published:** 2017-02-16

**Authors:** Sarah Lindgren Belal, May Sadik, Reza Kaboteh, Nezar Hasani, Olof Enqvist, Linus Svärm, Fredrik Kahl, Jane Simonsen, Mads H. Poulsen, Mattias Ohlsson, Poul F. Høilund-Carlsen, Lars Edenbrandt, Elin Trägårdh

**Affiliations:** 1 Department of Translational Medicine, Lund University, Malmö, Sweden; 2 Department of Clinical Physiology, Sahlgrenska University Hospital, Göteborg, Sweden; 3 Department of Signals and Systems, Chalmers University of Technology, Göteborg, Sweden; 4 Eigenvision AB, Malmö, Sweden; 5 Department of Nuclear Medicine, Odense University Hospital, Odense, Denmark; 6 Department of Urology, Odense University Hospital, Odense, Denmark; 7 Department of Astronomy and Theoretical Physics, Lund University, Lund, Sweden

**Keywords:** PET/CT, Sodium fluoride, Bone scan index, Imaging biomarker, Prostate cancer

## Abstract

**Background:**

Sodium fluoride (NaF) positron emission tomography combined with computer tomography (PET/CT) has shown to be more sensitive than the whole-body bone scan in the detection of skeletal uptake due to metastases in prostate cancer. We aimed to calculate a 3D index for NaF PET/CT and investigate its correlation to the bone scan index (BSI) and overall survival (OS) in a group of patients with prostate cancer.

**Methods:**

NaF PET/CT and bone scans were studied in 48 patients with prostate cancer. Automated segmentation of the thoracic and lumbar spines, sacrum, pelvis, ribs, scapulae, clavicles, and sternum were made in the CT images. Hotspots in the PET images were selected using both a manual and an automated method. The volume of each hotspot localized in the skeleton in the corresponding CT image was calculated. Two PET/CT indices, based on manual (manual PET index) and automatic segmenting using a threshold of SUV 15 (automated PET_15_ index), were calculated by dividing the sum of all hotspot volumes with the volume of all segmented bones. BSI values were obtained using a software for automated calculations.

**Results:**

BSI, manual PET index, and automated PET_15_ index were all significantly associated with OS and concordance indices were 0.68, 0.69, and 0.70, respectively. The median BSI was 0.39 and patients with a BSI >0.39 had a significantly shorter median survival time than patients with a BSI <0.39 (2.3 years vs not reached after 5 years of follow-up [*p* = 0.01]). The median manual PET index was 0.53 and patients with a manual PET index >0.53 had a significantly shorter median survival time than patients with a manual PET index <0.53 (2.5 years vs not reached after 5 years of follow-up [*p* < 0.001]). The median automated PET_15_ index was 0.11 and patients with an automated PET_15_ index >0.11 had a significantly shorter median survival time than patients with an automated PET_15_ index <0.11 (2.3 years vs not reached after 5 years of follow-up [*p* < 0.001]).

**Conclusions:**

PET/CT indices based on NaF PET/CT are correlated to BSI and significantly associated with overall survival in patients with prostate cancer.

## Background

Bone is the most frequent site of metastases in prostate cancer, and the standard imaging technique for detection of bone involvement is two-dimensional (2D) whole-body bone scan [1]. The bone scan index (BSI), obtained from planar whole-body bone scans, is the first quantitative imaging biomarker in prostate cancer and constitutes a surrogate for the tumor burden which is presented as a percentage of the total skeletal mass. The development of automatically calculated BSI has markedly reduced the interpretation time and decreased inter-observer variability compared to visual analysis alone [2, 3]. Several studies have confirmed that automated BSI has standardized the calculation of BSI and represents a consistent imaging biomarker for patients with advanced prostate cancer. Automated BSI provides clinicians with prognostic information as it is an independent predictor of survival, and can assess response to therapy in men with metastasized prostate cancer [4–7].

Positron emission tomography (PET) combined with computed tomography (CT) is a rapidly growing imaging modality and its role in oncologic diagnostics has expanded during recent years. Unlike planar bone scan, PET/CT is a three-dimensional (3D) method that can quantitatively assess biologic processes using specific radiotracers such as ^18^F-fluorodeoxyglucose, ^11^C-acetate, ^11^C-choline, ^18^F-sodium fluoride (NaF), and ^68^Ga-prostate-specific membrane antigen. NaF has specific affinity for bone and can be used to track skeletal pathology. Several studies have indicated that NaF PET/CT has superior sensitivity compared to bone scan in detecting skeletal changes due to bone metastasis in prostate cancer [8–10]. However, the interpretation of NaF PET/CT still poses a challenge. Similar to bone scan interpretation prior to the development of BSI, there is no objective method to evaluate skeletal uptake in PET/CT scans. The prostate cancer working group 3 consensus criteria state that there is a lack of standards in NaF PET interpretation for reporting disease presence or changes posttreatment and that NaF should be approached as a new biomarker subjected to independent validation [11]. Quantification from NaF PET/CT images could make it possible to stratify prognosis and track disease progress. It would also yield an objective way of evaluating treatment outcome which would enable the development of new therapies.

The aim of this study was to develop a 3D PET/CT index which reflects tracer uptake due to tumor burden in the skeleton in a similar way as BSI. A secondary aim was to compare PET/CT index to BSI in the same group of patients with prostate cancer and the association between PET/CT index, BSI, and overall survival (OS).

## Methods

### Training group

The automated segmentation of the skeleton in the CT images was developed using a retrospective training set from 25 patients who had undergone PET/CT examinations between 2008 and 2010 at Sahlgrenska University Hospital, Gothenburg, Sweden. The study was conducted according to the principles expressed in the Declaration of Helsinki, approved by the local research ethics committee at University of Gothenburg (# 295-08), and informed consent was obtained from each subject.

### Study group

We retrospectively studied PET/CT scans and bone scans in prostate cancer patients who previously had been selected for a study at Odense University Hospital, Denmark, with the aim to compare whole-body bone scans, choline-PET/CT, and NaF PET/CT with MRI [12]. The inclusion criteria in that study were (1) biopsy-proven prostate cancer, (2) a current bone scan with a minimum of one metastasis, (3) the ability to undergo MRI, and (4) the ability to safely postpone treatment with androgen deprivation until after all scans were finalized. The exclusion criteria were (1) current or previous treatment with androgen deprivation, and (2) pain or suspicion of spinal cord compression based on malignant bone lesions. Bone scans, PET/CT scans, and MRI were performed within a time frame of 1 month in random order. A total of 50 patients, aged 53–92 years, were included between May 2009 and March 2012.

For the current study, only bone and NaF PET/CT scans were utilized. Staging information, i.e., PSA values and Gleason score, was collected. Dates for all scans and survival data were collected from the local radiology information system. The study was conducted according to the principles expressed in the Declaration of Helsinki, approved by the local research ethics committees at Lund University (# 2016/193) and Odense University Hospital (# 3-3013-1692/1).

### Image acquisition

#### Training group

PET/CT data were obtained using an integrated PET/CT system (Siemens Biograph 64 Truepoint). A low dose CT scan (64-slice helical, 120 kV, “smart mA” maximum 30-110 mA) was obtained from the base of the skull to the mid-thigh. The CT slice thickness used in the analysis was 3.27 mm.

#### Study group

PET/CT data were obtained by a Discovery VCT PET/CT scanner (GE Healthcare). All patients received an injection of 3 MBq NaF per kg body weight after having fasted for 6 h. Image acquisition started approximately 60 min after tracer injection. A diagnostic contrast-enhanced CT scan (64-slice helical, 120 kV, “smart mA” maximum 400 mA) was obtained from the base of the skull to the mid-thigh. The CT slice thickness used in the analysis was 3.75 mm. A PET scan with an acquisition time of 2.5 min per bed position was obtained from the same region.

Whole-body planar bone scans with anterior and posterior views were acquired using a dual head ɣ camera (Skylight or PRISM XP2000, Philips Medical, Surrey) with LEHR collimator, energy window 140 keV ±20%, matrix 256×1024, and scan speed 14 cm/min. All patients received 600 MBq Tc-99m HDP and imaging acquisition was performed 3 h postinjection.

### Bone scan index

EXINIbone^BSI^ version 2 (EXINI Diagnostics AB, Lund, Sweden) was used to analyze the bone scans and automatically generates the BSI data. Manual corrections were made according to the manufacturer’s instructions, i.e., if a hotspot was included in the BSI calculation, but clearly represented known trauma, urinary bladder, urinary bag/catheter, or site of injection, it was excluded from the BSI calculation. Other hotspots were not re-classified.

The methodology of the automated platform has been described in detail in previous studies [3]. In summary, the different anatomical regions of the skeleton are segmented followed by detection and classification of abnormal hotspots as metastatic lesions. The fraction of the skeleton for each metastatic hotspot is calculated and the BSI is calculated as the sum of all such fractions.

### PET/CT index

1. Segmentation of skeleton
  Step 1: Convolutional neural network-based landmark detection
    A convolutional neural network [17, 18] was trained to detect a number of anatomical landmarks, and a second network to detect center lines for the humeri, ribs, clavicles, and femurs (Fig. 1).

**Fig. 1:**
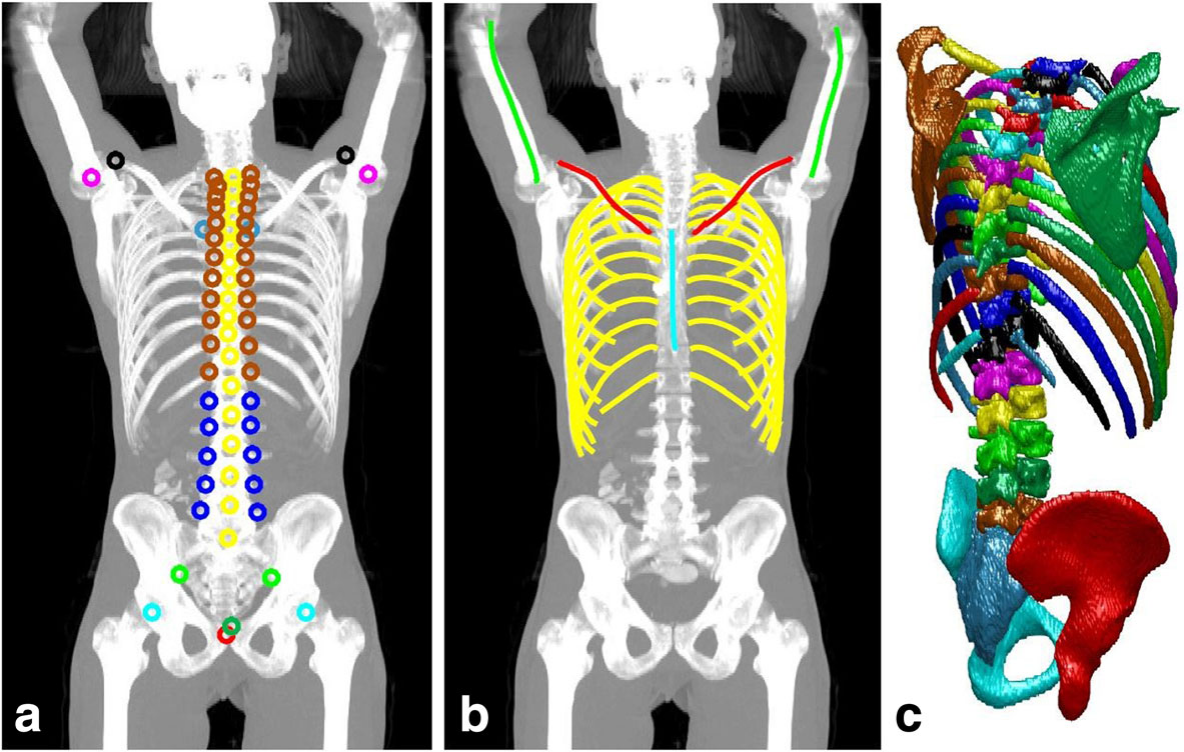
**a** Maximum intensity projection of the CT scan together with the annotated landmarks. Landmarks with *identical markers* belong to the same class and are not separated by the detector. **b** Detected *center lines* for ribs, clavicles, and humeri. **c** Surface reconstruction of the resulting segmentation. This underlying image belongs to the test set and has not been involved in training the neural networks
  Step 2: Geometric model fitting
    Partly due to the limited training set, the convolutional neural network-based detectors produced a number of false positives but very few false negatives. To handle this, geometric models were used to prune false landmark detections and determine rough positions for the relevant anatomical structures. Essentially two types of models were used. The first was an iterative technique to track elongated bones such as ribs and clavicles. The second type was a classical active shape models used to find plausible positions for groups of landmarks.
  Step 3: Convolutional neural network-based pixel-wise segmentation
    The final step of the automated segmentation technique was the application of another convolutional neural network trained to perform pixel-wise segmentation of the CT image. The input to the network was not only the CT image but also a second channel with a rough segmentation based on an atlas registered using the aligned landmarks.
  An automated segmentation of the following bones was performed in the CT scans: The thoracic and lumbar spines, sacrum, pelvis, ribs, scapulae, clavicles, and sternum. The slice thickness of the CT images of 3-4 mm made it difficult to segment the cervical vertebrae and they were therefore not included. In addition, the skull, humeral, femoral, and other appendicular bones were not segmented since they were not always completely included in the CT scans. A total of 49 bones were segmented, comprising approximately 33% of the total skeletal volume [13].
  The automated segmentation method was developed using the separate training set of CT scans. Three experienced readers manually segmented the skeleton in these CT scans using the TurtleSeg software [14–16]. After the training process, the automated method was applied to the CT scans of the study group. The segmentation process can be divided into three steps:
2. Hotspot detection and classification
  Volumes in the PET images with uptake above a given standard uptake value (SUV) were defined as hotspots. Two separate methods were used to select this given SUV value and hotspots for inclusion in the PET/CT index.
  Manual: With this method, we aimed to reflect the clinical interpretations of the PET/CT scans as closely as possible. For each individual patient first, an optimal SUV threshold for detection of hotspots was selected, based on the visual interpretation of a nuclear medicine specialist who was blinded to the patients’ bone scans, BSI values, and survival data. The choice of threshold was made so that all hotspots interpreted as caused by metastatic disease by the nuclear medicine specialist were delineated. After selecting a threshold, each detected hotspot was manually classified as caused by metastatic disease or not, based on the interpretation of the nuclear medicine specialist. Hotspots believed to originate from degeneration, inflammation, or fractures were excluded from the analysis. Selected thresholds ranged between SUV 6–9.
  Automated: In a completely automated method, a SUV threshold of 15 was used to detect hotspots. This threshold was used in a recent study by Lin et al [19]. No manual selection was done.
  To avoid an unmanageable number of hotspots, smoothing with a Gaussian filter (standard deviation 2 mm) was performed before defining the hotspots. Hotspots that had no overlap with the segmented bone from the CT scans were removed.
3. PET/CT index calculation
  The volume of each hotspot classified as metastasis and localized in the skeleton in the corresponding CT scan was calculated. A PET/CT index was then calculated by dividing the sum of all such hotspots with the volume of the segmented bones, i.e., the thoracic and lumbar spines, sacrum, pelvis, ribs, scapulae, clavicles, and sternum. Two indices were calculated from each patient’s PET/CT scan: one based upon the manual method (manual PET index) and one based upon the automated method using the SUV threshold of 15 (automated PET_15_ index).
  The BSI is defined as the fraction of the total skeleton that is involved by tumor, and skeletal parts not included in the analysis were assumed to have no metastases. Accordingly, both PET/CT indices were multiplied by 0.33 since the bones included in the PET/CT indices comprised 33% of the total skeletal volume [13].

### Statistical analyses

Overall survival was defined as time from NaF PET/CT and bone scan to death/follow-up, respectively. Cutoff date for analysis was October 28, 2016. Kaplan-Meier estimates and the log-rank test were used to estimate the survival difference between high and low BSI and PET/CT index groups. The group with high indices was defined as those with values above the median value and the group with low indices as those with values below the median value. The choice of a median split was made as there are no previous studies on the PET/CT index. A *p* value <0.05 was considered significant. In the survival analysis, all data were censored at a follow-up after 5 years.

The association between the different indices and OS was evaluated using a univariate Cox proportional hazards regression model. Hazard ratios (HR) together with 95% confidence intervals (CI) were estimated, and the performance assessment of the different survival models was measured using the concordance index (C-index). The difference in C-indices between different models was assessed using the method described by Haibe-Kains et al [20]. The Bland-Altman method was used to assess the agreement between the different indices. All analyses were carried out using R statistical computing environment [21] and IBM SPSS Statistics 24.

## Results

Forty-eight of the 50 patients in the study group had both a bone scan and a NaF PET/CT available for quantitative analysis, while in two patients, the technical quality of the images was not sufficient for the retrospective quantitative analysis. Patient characteristics for the 48 patients are presented in Table 1.

**Table 1:** Patient characteristics

	Mean (SD)	Median (range)	Number of patients
Age (years)	73 (8.6)	73 (53–92)	48
PSA (μg/L)	374 (874)	84 (4–5740)	48
Gleason score	7.7 (1.5)	8.0 (5–10)	47

The 48 patients had a median observation time of 3.7 years (interquartile range [IQR] 1.9–6.0 years) after NaF PET. A total of 34 patients died during the follow-up period, with a median survival time from the baseline NaF PET of 2.4 years (IQR 1.5–3.6). The group of 14 men that were still alive had a median follow-up time from the baseline NaF PET of 6.2 years (IQR 5.7–6.9).

**Fig. 2:**
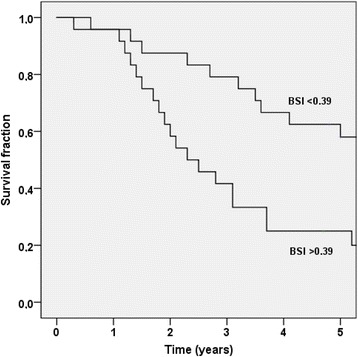
The Kaplan-Meier survival curves for the two BSI groups (BSI <0.39 and BSI >0.39)

The median BSI was 0.39 (IQR 0.08–2.05). The patients with a BSI >0.39 had a significantly shorter median survival time than patients with a BSI <0.39 (2.3 years vs not reached after 5 years of follow-up (*p* = 0.01)). Figure 2 shows the Kaplan-Meier survival curves for these two groups. BSI was significantly associated with OS in a univariate Cox analysis (HR 1.26, 95% CI 1.13–1.41; *p* < 0.001) and the C-index was 0.68 (95% CI 0.59–0.76).

**Fig. 3:**
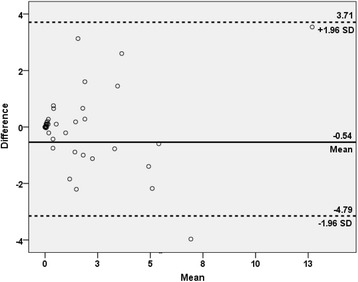
The Bland–Altman plot of the difference between BSI and manual PET index against the mean of BSI and manual PET index

**Fig. 4:**
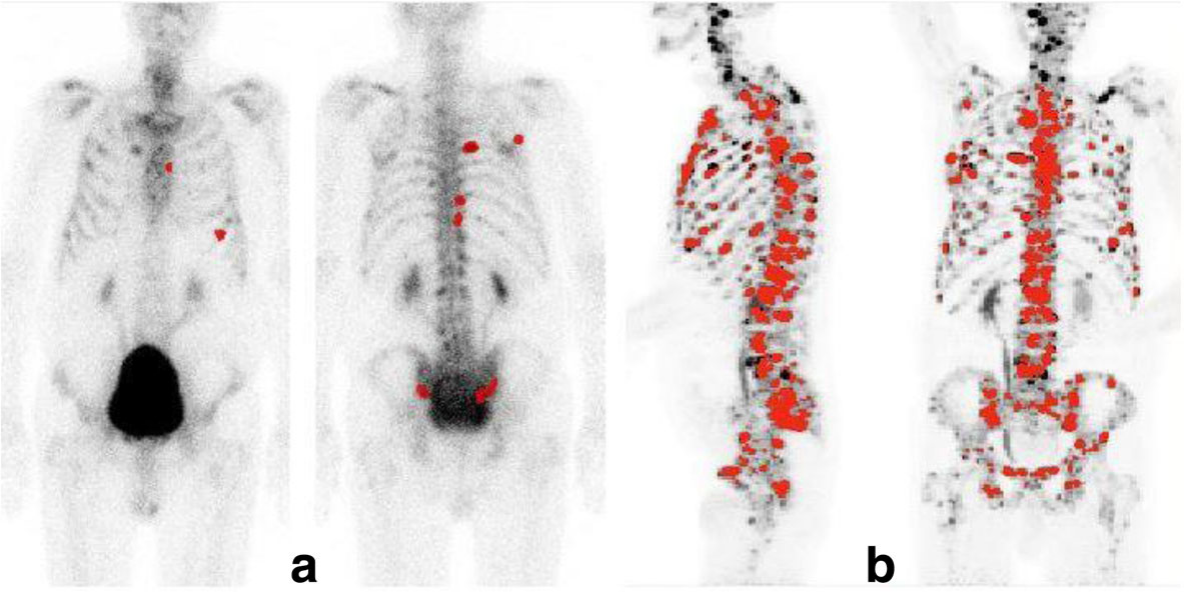
Patient example showing hotspot segmentation in **a** bone scan (anterior and posterior views) with a BSI of 0.4% and **b** maximum intensity projection NaF PET/CT scans with a PET index of 2.6%. Note that the BSI analysis is based on the two images showed in (**a**) whereas the PET/CT indices are based on a 3D analysis and not the two projection images showed in this figure

**Fig. 5:**
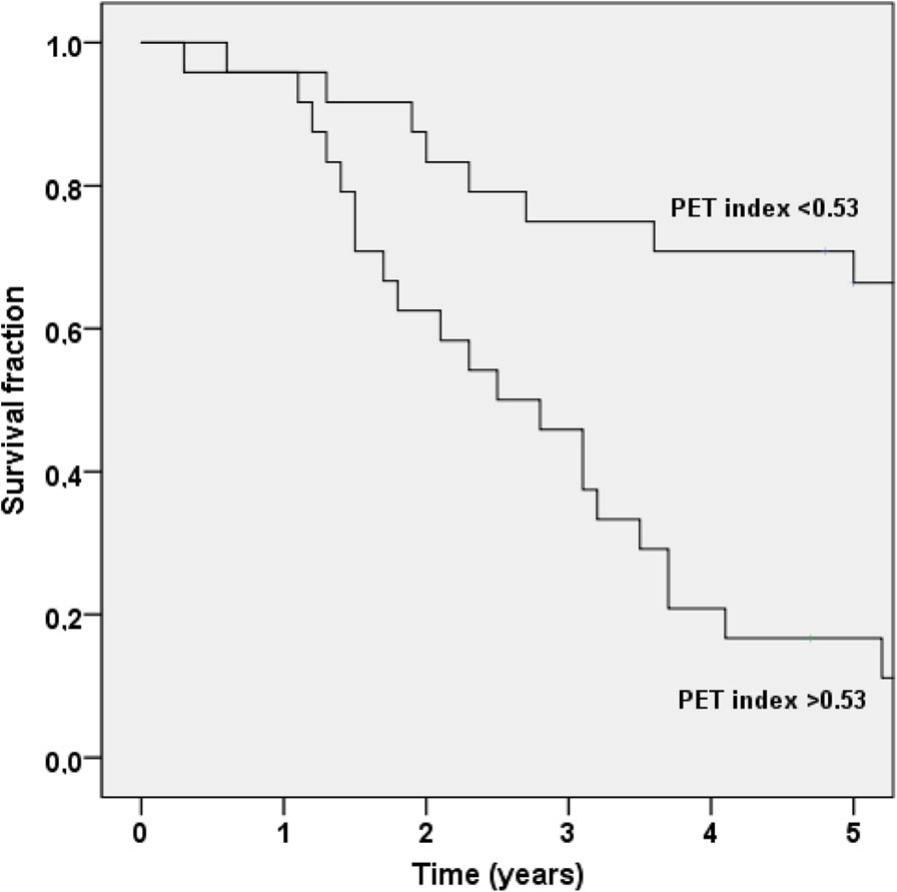
The Kaplan-Meier survival curves for the two manual PET index groups (index <0.53 and >0.53)

The correlation between the manual PET index and BSI is plotted in Fig. 3. The most common divergence between the indices was a higher manual PET index than BSI, exemplified by the patient in Fig. 4. The median manual PET index was 0.53 (IQR 0.02–2.62). The patients with a manual PET index >0.53 had a significantly shorter median survival time than patients with a manual PET index <0.53 (2.5 years vs not reached after 5 years of follow-up [*p* < 0.001]). Figure 5 shows the Kaplan-Meier survival curves for these two groups. The manual PET index was significantly associated with OS in a univariate Cox analysis (HR 1.17, 95% CI 1.06–1.29; *p* = 0.002) and C-index was 0.69 (95% CI 0.60–0.78).

**Fig. 6:**
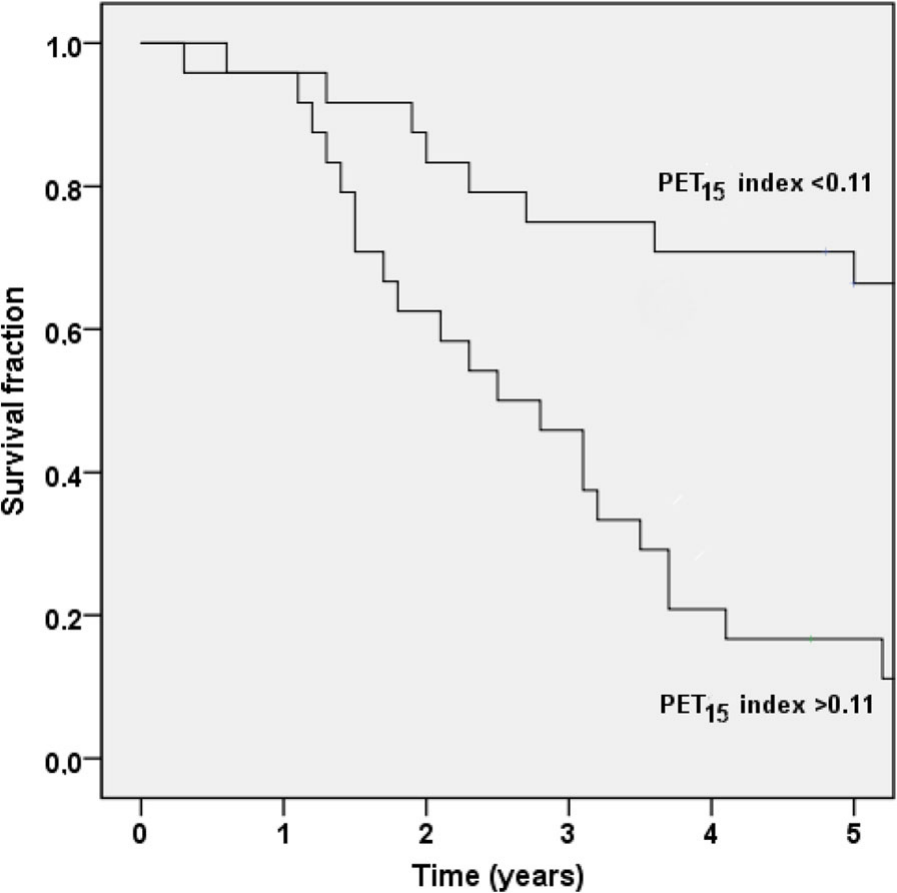
The Kaplan-Meier survival curves for the two automated PET_15_ index groups (index <0.11 and >0.11)

**Fig. 7:**
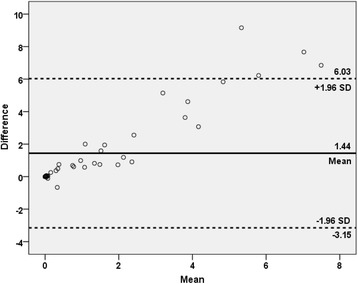
The Bland–Altman plot of the difference between manual PET index and automated PET_15_ index against the mean of manual PET index and automated PET_15_ index

The median automated PET_15_ index was 0.11 (IQR 0.00–0.98). The patients with an automated PET_15_ index >0.11 had a significantly shorter median survival time than patients with an automated PET_15_ index <0.11 (2.3 years vs not reached after 5 years of follow-up [*p* < 0.001]). Figure 6 shows the Kaplan-Meier survival curves for these two groups. The automated PET_15_ index was also significantly associated with OS in a univariate Cox analysis (HR 2.01, 95% CI 1.43–2.83; *p* < 0.001) and C-index was 0.70 (95% CI 0.61–0.79) (Table 2). The automated PET_15_ index was lower than the manual PET index in 39/48 patients. The average automated PET_15_ index was 0.7 and the average manual PET index was 2.1, i.e., only approximately 1/3 of the tumor burden as defined in the visual interpretation was reflected in the PET_15_ index. The relation between these two indices is presented in Fig. 7.

**Table 2:** C-index and univariate Cox regression analysis (*N* = 48)

	C-index	95% CI	*p* value	Hazard ratio	95% CI	*p* value
BSI	0.68	0.59–0.76	<0.001	1.26	1.13–1.41	<0.001
PET index	0.69	0.60–0.78	<0.001	1.17	1.06–1.29	=0.002
PET_15_ index	0.70	0.61–0.79	<0.001	2.01	1.43–2.83	<0.001

The differences in C-index between BSI and manual PET index, BSI and automated PET_15_ index, and manual PET index and automated PET_15_ index were not statistically significant (*p* = 0.60, 0.89, and 0.75, respectively).

## Discussion

### Main results

In this preliminary study, we have shown that PET/CT indices based on NaF PET/CT scans, which reflects similar processes in the bone of prostate cancer patients as BSI, are significantly associated with OS in a group of prostate cancer patients. The result for the association between baseline BSI and survival is in agreement with previous studies [3, 22].

NaF PET/CT scans have shown to be more sensitive than bone scans in detecting bone changes due to metastases, but a disadvantage has been the lack of a quantitative method to evaluate pathological skeletal uptake in PET/CT scans. In this study, two different PET/CT indices were studied; one aimed to reflect visual interpretation by a nuclear medicine specialist, and one automatically generated. The higher sensitivity of NaF PET/CT compared to bone scan was reflected by higher manual PET index than BSI being more common than the opposite finding, and a slightly but not significantly higher C-index. Future studies are needed to evaluate the possible increased clinical value of a PET/CT index versus BSI.

Quantitative measurements need to be reproducible and objective in order to qualify as an imaging biomarker. An automated method can be validated analytically and clinically and is not dependent on the knowledge and experience of the interpreting reader. BSI calculation using EXINIbone^BSI^ is an objective fully automated approach to quantify skeletal tumor burden in bone scans. The aim of our research is to develop an automated PET/CT index using methods similar to those used for BSI calculations. Methods of these types require training databases of scans to mimic interpretation by experts. In this study, such a training database was not available and we therefore studied an automated PET_15_ index, which was based on a SUV threshold of 15. This SUV threshold has been used in a recent publication by Lin et al. to exclude hotspots with low statistical likelihood of being metastases [19]. A disadvantage with this automated PET_15_ index was that it reflected on average only 1/3 of the tumor burden as defined in the visual interpretation were thresholds ranged between SUV 6–9. We will therefore continue to develop an automated method that more closely reflects the results of visual interpretation.

There is relatively little data on how to differ metastatic from non-metastatic uptake in NaF PET/CT based on SUV. It is therefor unclear what threshold for automatic hotspot identification and segmentation that is optimal in order to generate hotspots that best reflect true tumor burden. Based on our results, using SUV 15 as a threshold for automatic hotspot segmentation reflects less tumor burden than BSI, despite the higher sensitivity of NaF PET/CT compared to bone scan. This may indicate that using a threshold of SUV 15 may lead to exclusion of hotspots that are metastatic origin. We will continue to investigate thresholds for hotspot segmentation. Also, different ways to automatically delineate hotspots, leading to different hotspot volumes and thus different PET/CT indices, will be further studied. Other features to identify hotspots with suspected metastatic origin may also be investigated, such as different locations within the bone, which could help to differentiate between metastases and degenerative changes.

### Limitations

Fluoride accumulation in PET/CT scans is not specific for metastatic activity. Fluoride is incorporated in the bone as hydroxyapatite, forming fluoroapatite and fluorohydroxyapatite, and activity increases as a sign of osteoblastic activity [1, 23]. Focal uptake can represent other causes of increased bone turnover, such as degeneration, fractures in healing, or inflammation. In addition, focal bone changes may persist for quite some time after effective cancer therapy and through that give a false impression of the degree of malignant bone involvement [23–25]. Hence, the pharmacokinetic radiotracer uptake is an inherent limitation in NaF PET/CT scans in the same way as in bone scans.

### Clinical implications

There is a clinical need for a quantitative and a reproducible assessment of tumor burden in metastatic prostate cancer patients. BSI has shown to be a valuable imaging biomarker with clinical relevance in this patient group. A high BSI is associated with a poor prognosis both at the time of diagnosis and at more advanced stages of the disease [26–28], and an increase in BSI during treatment signals worse outcome than if BSI remains stable or decrease during therapy [4, 29, 30]. The same quantitative approach applied to NaF PET/CT scans would most likely be successful since the superior performance of NaF PET/CT compared to planar bone scans is well documented [11, 12]. If done in an automated fashion, it could decrease intra-observer variability and help physicians to assess disease progress or response to therapy, thereby affecting clinical decisions [2]. Although it is encouraging that both manual PET index and automated PET_15_ index were associated with OS in this preliminary study, it is too early to introduce such an index in clinical routine. We hope that further development of this method can result in an automated PET/CT index that can serve as an imaging biomarker with prognostic and predictive information in patients with prostate cancer.

## Conclusions

We have showed that the amount of increased focal skeletal uptake determined from NaF PET/CT scans is associated with OS in prostate cancer patients. A PET/CT index which reflects tracer uptake due to tumor burden to the skeleton in a similar way as BSI can be used to evaluate NaF PET/CT images in a quantitative way. This type of PET/CT index will most likely be of value both in a clinical settings and in future clinical trials.

## Abbreviations

2D: Two-dimensional
3D: Three-dimensional
BSI: Bone scan index
C-index: Concordance index
CT: Computed tomography
HR: Hazard ratio
IQR: Interquartile range
NaF: Sodium fluoride
OS: Overall survival
PET: Positron emission tomography
SUV: Standard uptake value
