# Correction to: 3D skeletal uptake of ^18^F sodium fluoride in PET/CT images is associated with overall survival in patients with prostate cancer

**DOI:** 10.1186/s13550-019-0510-0

**Published:** 2019-05-20

**Authors:** Sarah Lindgren Belal, May Sadik, Reza Kaboteh, Nezar Hasani, Olof Enqvist, Linus Svärm, Fredrik Kahl, Jane Simonsen, Mads H. Poulsen, Mattias Ohlsson, Poul F. Høilund-Carlsen, Lars Edenbrandt, Elin Trägårdh

**Affiliations:** 1 Department of Translational Medicine, Lund University, Malmö, Sweden; 2 Department of Clinical Physiology, Sahlgrenska University Hospital, Göteborg, Sweden; 3 Department of Signals and Systems, Chalmers University of Technology, Göteborg, Sweden; 4 Eigenvision AB, Malmö, Sweden; 5 Department of Nuclear Medicine, Odense University Hospital, Odense, Denmark; 6 Department of Urology, Odense University Hospital, Odense, Denmark; 7 Department of Astronomy and Theoretical Physics, Lund University, Lund, Sweden

**Correction to: EJNMMI Res**

**https://doi.org/10.1186/s13550-017-0264-5**

Unfortunately, the original version of this article contains an error. In Fig. 6, the plotted curves are incorrect. Please note that the original data is correct and statistical tests are valid for the survival analysis. The correct version of Fig. 6 can be found below.

**Fig. 6:**
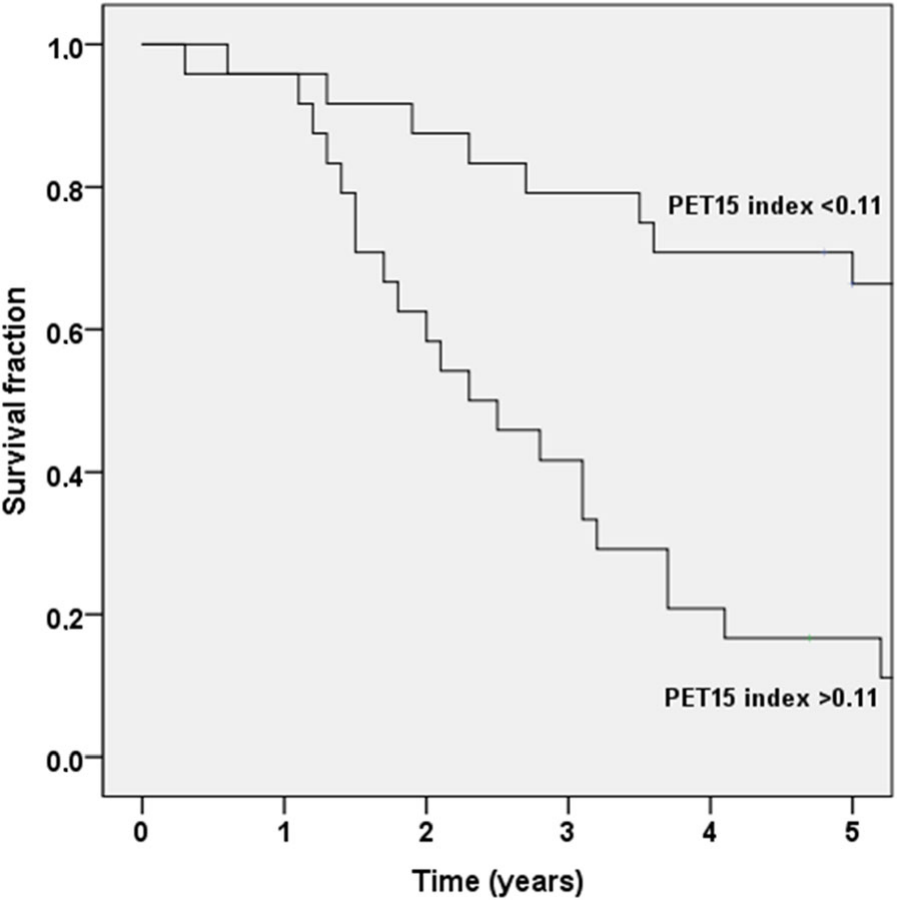
The Kaplan-Meier survival curves for the two automated PET_15_ index groups (<0.11 and >0.11)
